# Correction: Tamayo et al. Botulinum Toxin in Pain-Related Post-Stroke Limb Spasticity: A Meta-Analysis of Early and Late Injections. *Toxins* 2025, *17*, 258

**DOI:** 10.3390/toxins18050202

**Published:** 2026-04-27

**Authors:** Frances Marie Tamayo, Raymond Rosales, Jörg Wissel, Bo Biering-Sørensen, Joshua Nathaniel Ellano, David Simpson

**Affiliations:** 1 Department of Neuroscience and Behavioral Medicine, University of Santo Tomas Hospital, Manila 1015, Philippines; 2 Research Center for Health Sciences, Faculty of Medicine and Surgery, University of Santo Tomas, Manila 1015, Philippines; 3 Department of Neurorehabilitation and Physical Therapy, Vivantes Hospital Spandau, 13585 Berlin, Germany; wissel@rehaklinik-beelitz.de; 4 NeuroScience Centre, Rigshospitalet, Copenhagen University Hospital, Havnevej 25, DK-3100 Hornbæk, Denmark; bo.biering-soerensen@regionh.dk; 5 Department of Nursing, College of Nursing, University of Santo Tomas, Manila 1015, Philippines; nathaniel.ellano@gmail.com; 6 Department of Neurology, Icahn School of Medicine at Mount Sinai, New York, NY 10029, USA; david.simpson@mssm.edu

In the original publication of this article [1], the authors stated that the review was registered in the PROSPERO database. Following publication, it was brought to our attention that the registration was not completed prior to commencement.

Therefore, the authors wish to remove the following text from the “Methods” section: “This meta-analysis was registered on PROSPERO, an international platform of registered systematic review and meta-analysis protocols (Prospero ID number: 1044077).”

The authors state that the scientific conclusions are unaffected. This correction was approved by the Academic Editor. The original publication has also been updated.
